# The effects of added sugar intake on cardiometabolic health and memory recall in midlife adults

**DOI:** 10.1002/alz.095601

**Published:** 2025-01-09

**Authors:** Kevin P. Decker, Zoe Rigas, Nicholas A. Rizzi, Catherine Awad, Faria Sanjana, Matthew L. Cohen, Shannon L. Lennon, Christopher R. Martens

**Affiliations:** ^1^ University of Delaware, Newark, DE USA

## Abstract

**Background:**

Aging is the primary risk factor for Alzheimer’s disease (AD) which is the most common cause of dementia. The risk factors for AD emerge during midlife and are similar to cardiometabolic diseases. Midlife cardiometabolic changes are worsened by poor lifestyle habits, such as consuming a Western Diet (WD), which is partially characterized by high added sugar intake (i.e., all caloric sweeteners added to food during cooking or processing). This study aimed to investigate if short‐term consumption of excessive added sugars acutely alters cardiometabolic risk factors and hippocampal‐dependent memory function in midlife adults (50‐64 years old). We hypothesized that, compared to a low added sugar diet, the high added sugar diet would be detrimental to cardiometabolic and brain health.

**Method:**

In a randomized order, 26 participants (10 males / 16 females) were assigned to consume a 10‐day high‐added sugar (HS: 25% total calories) and low‐ added sugar (LS: 5% total calories) diet. At the end of each diet, blood was sampled, blood pressure was measured, and memory recall was tested using the Hopkins Verbal Learning Test (HVLT) and the Brief Visuospatial Memory Test (BVMT).

**Result:**

Compared to the LS diet, the HS diet significantly increased plasma triglycerides (LS: 91±31; HS: 102±30 mg/dL; p = 0.04) and mean arterial blood pressure (LS: 80±7; HS: 85±9 mmHg; p = 0.002). After the HS diet, total memory recall scores were significantly lower for BVMT: (LS: 26.0±5.2; HS 23.3±5.9 correct responses; p = 0.02) but not the HVLT. Compared to the LS diet, delayed memory recall scores were significantly lower after the HS diet for both tests [(HVLT: (LS: 10.5±1.5; HS 9.9±1.7 correct responses; p = 0.03) and BVMT: (LS: 10.1±1.6; HS 8.8±2.2 correct responses; p = 0.002)].

**Conclusion:**

A short‐term (10‐day) diet with an excessive amount of added sugars increases cardiometabolic risk factors, whichmay make the brain susceptible to lower memory recall in otherwise healthy midlife adults. This study highlights the importance of diet on cardiometabolic and brain health in midlife adults. Future studies should examine the long‐term impact of added sugars on AD risk and explore the underlying mechanisms by which added sugars contribute to cognitive aging.

